# Financial Impact of Inaccurate Coding Plus Cost-Effectiveness Analysis for Surgically Managed Patients With Periprosthetic Fractures

**DOI:** 10.7759/cureus.13060

**Published:** 2021-02-01

**Authors:** Ethan Toner, Ahmad Khaled, Ashwanth Ramesh, Mobeen K Qureshi, Kais Al Suyyagh, Paul Dunkow

**Affiliations:** 1 Trauma and Orthopaedics, Royal Victoria Hospital, Belfast, GBR; 2 Trauma and Orthopaedics, Blackpool Victoria Hospital, Blackpool, GBR; 3 Trauma and Orthopaedics, Lancaster General Hospital, Lancaster, GBR; 4 Trauma and Orthopaedics, Royal Bolton Hospital, Bolton, GBR

**Keywords:** medical coding, periprosthetic fractures, cost effective analysis

## Abstract

Background

An upward trend is seen in a number of periprosthetic fractures. Their management often requires complex surgical intervention, expert skills and expensive equipment. Hospitals get paid according to Healthcare Resource Group (HRG) tariffs. HRG gets generated once diagnoses, Charlson comorbidity (CC) index score, surgical procedures, investigations and length of stay have been coded for. Coding departments consist of non-clinicians. Although auditing systems are in and made of internal and external auditors, we hypothesized that multiple errors can still occur which may result in significant financial losses.

Objectives

To assess the accuracy of coding for management of periprosthetic fractures. To identify causes for inaccurate coding and assess the financial impact of highly complex trauma in a district general hospital (DGH).

Methods

Retrospective comparative analysis of case notes for patients with an M966 diagnosis code (periprosthetic fracture) between 1st November 2017 and 1st November 2018. All cases were analysed and data for primary procedure, primary diagnosis, secondary procedures and secondary diagnosis, comorbidities and length of stay were extrapolated and re-coded using the same software in use by the coding team. Costs incurred for each surgically managed patient were calculated using a rough estimate of cost of each procedure. Finally, cost-effectiveness analysis was carried out by comparing our calculated figures to the actual final claim by our institution.

Results

Twenty-nine patients with the diagnosis of periprosthetic fracture were identified by the coding team using M966 code. A further case was identified by reviewing operating software (Operating Room Management Information System [ORMIS®]). In four cases (13.3 %), the primary diagnosis was coded incorrectly by the coding team. Overall coders accuracy for surgically managed patients (n=21) was 52% (n=11). This resulted in an estimated incurred loss of £25,000. Wrong/omitted site of surgery was found to be the most influential coder error with up to £8000 loss in one case (P<0.05). Cost-effectiveness analysis demonstrated the stark differences in costs for HRG tariffs when used in trauma setting vs non-trauma setting. Open reduction and internal fixation (ORIF) was associated with less financial loss to our trust with closer procedural costs to HRG tariff (average cost of £9200 for ORIF vs £22,030 for a massive endoprosthesis).

Conclusions

Surgeons should carefully review codes for such complex procedures before or soon after surgery. Wrong/omitted site of surgery is the key cause for losses in our cohort, followed by inadequate recording of comorbidities. Coders can only code for what is documented. Following cost-effectiveness analysis our study highlights the need for HRG tariffs to be revised for such procedures. The cost of ORIF vs massive endoprosthesis should be noted, signifying the implant costs when such specialised revision surgery performed over less expensive ORIF surgery.

## Introduction

Periprosthetic fractures are becoming a common presentation to trauma units throughout the UK [1]. Combined factors of an aging population along with increased arthroplasty and hemiarthroplasty surgery performed worldwide, suggest an increasing upward trend in the number of periprosthetic fractures.

The latest 2019 report of national joint registries worldwide states the incidence of periprosthetic fractures to total hip replacement is 23.6% over the last five years [2]. Periprosthetic fractures incidence has been predicted to be as high as 6.9% in 2020 [1]. Management of periprosthetic fractures is highly complex requiring planned surgery, high level of technical skills, and expensive implants and instruments.

Within the National Health Service (NHS) certain procedures are said to consume the same amount of health resources and are therefore often grouped together into Healthcare Resource Groups (HRG). The HRG tariff system is an activity payment-based system used to determine the income hospitals get paid for given procedures [3]. Each HRG covers a period of clinical care, from admission to discharge [3]. Upon discharge from hospital, an HRG code is generated for each patient visit using two classification systems, ICD-10 (International Statistical Classification of Diseases and related Health Problems) for diagnoses and OPCS-4 (Office of Population Censuses and Surveys) for interventions by clinical coders. HRG tariffs are calculated based on the diagnoses, Charlson Comorbidity Index (CC) score, surgical procedures, investigations and length of hospital stay [4]. 

There are around 26,000 HRG codes describing specific diagnoses and interventions [4]. Hospitals depend on their respective coding departments for the accuracy of coding and to ensure cost-effectiveness. Although auditing systems are in place consisting of internal and external auditors, coding errors can result in significant financial losses for the NHS.

Therefore, our aim was three-fold. Firstly, to assess accuracy of coding for the management of periprosthetic fractures. Secondly, to identify factors for inaccurate or incomplete coding. Thirdly, to assess the financial implications of coding errors in a large district general hospital.

## Materials and methods

A retrospective review of patient records was performed independently by the authors. Data were collected over a year, from November 2017 to 2018. Patients with periprosthetic fracture assigned with ICD-10 classification code of M966 were identified. Patient records were also cross-referenced with data from the Operating Room Management Information System (ORMIS^©^) to ensure all patients, in particular those who required surgical intervention during this period, were captured.

All data for primary procedure, primary diagnosis, secondary procedure, secondary diagnosis, co-morbidities and length of stay were extrapolated. All of the aforementioned parameters were re-inputted into the coding software in use in our institution to generate new HRG tariffs. The newly generated tariffs were then compared to the original tariffs submitted by the hospital coding team. When discrepancies were found, further analysis was carried out to look into the underlying causes for the coding error.

The CC index was developed to classify and risk-stratify patients with a weighted score for each of their comorbidities [5]. The index is used to predict mortality, measurement of disease burden and through coding algorithms to assist in administrative hospital discharge data (Table 1) [6].

**Table 1:** Charlson comorbidity index. Assigned weights for each condition that a patient has. The total equals the score. Example: chronic pulmonary (1) and lymphoma (2) = total score (3).

Assigned weights for disease	Conditions
1	Myocardial infarct
	Congestive heart failure
	Cerebrovascular disease
	Dementia
	Chronic pulmonary disease
	Connective tissue disease
	Ulcer disease
	Mild liver disease
	Diabetes
2	Hemiplegia
	Moderate or severe renal disease
	Diabetes with end organ damage
	Any tumour
	Leukemia
	Lymphoma
3	Moderate or severe liver disease
6	Metastatic solid tumour
	AIDS

Costs incurred for each surgically managed patient were calculated using a rough estimate of cost of each procedure (Table 2). Finally, cost-effectiveness analysis was carried out by comparing our calculated figures to the actual final claim by our institution. Patients or the public were not involved in the design, or conduct, or reporting, or dissemination plans of our research.

**Table 2:** Example of current coding and costs.

Current coding options for surgical management	Equipment and implant costing	Additional costs
Closed reduction and internal fixation	NCB Zimmer plates = £1800	Loan kits
Open reduction and internal fixation	Screws = £80-90	Theatre time (prolonged) = around £16.00 per minute
Bone grafting	Cables= £200-300 each	Drapes/procedure packs
Removal of implants	Explant kit (zimmer) = £500 loan	Pulse Lavage = £26
Conversion arthroplasty	Morelands (DePuy) = £190 loan	Aquacell dressings= £8
Revision arthroplasty	Oscar (Orthosonics)= £700	Saw blades
Endoprosthesis	PFR (Stryker) = £6000	Sutures
Massive endoprosthesis	PFR (Zimmer) = £20,000 (Currently stopped)	Gowns and gloves
	Dual mobility cup (Zimmer) = £900	
	Distal femoral replacement = £15000-20000	
	TC3 (Sleeves and Stem) = £12,000-£13,000	
	Bone graft = Tuto (£360/Box)	
	Hydroset (Stryker) = £800/box	
	Osteoset (Right Medical) = £460 for 20cc	
	Fresh frozen femoral head = £1000	
	Freeze dry Cortical Strut = £800	
	Corail stem= £900	
	Marathon Cup = £250	

## Results

**Figure 1:**
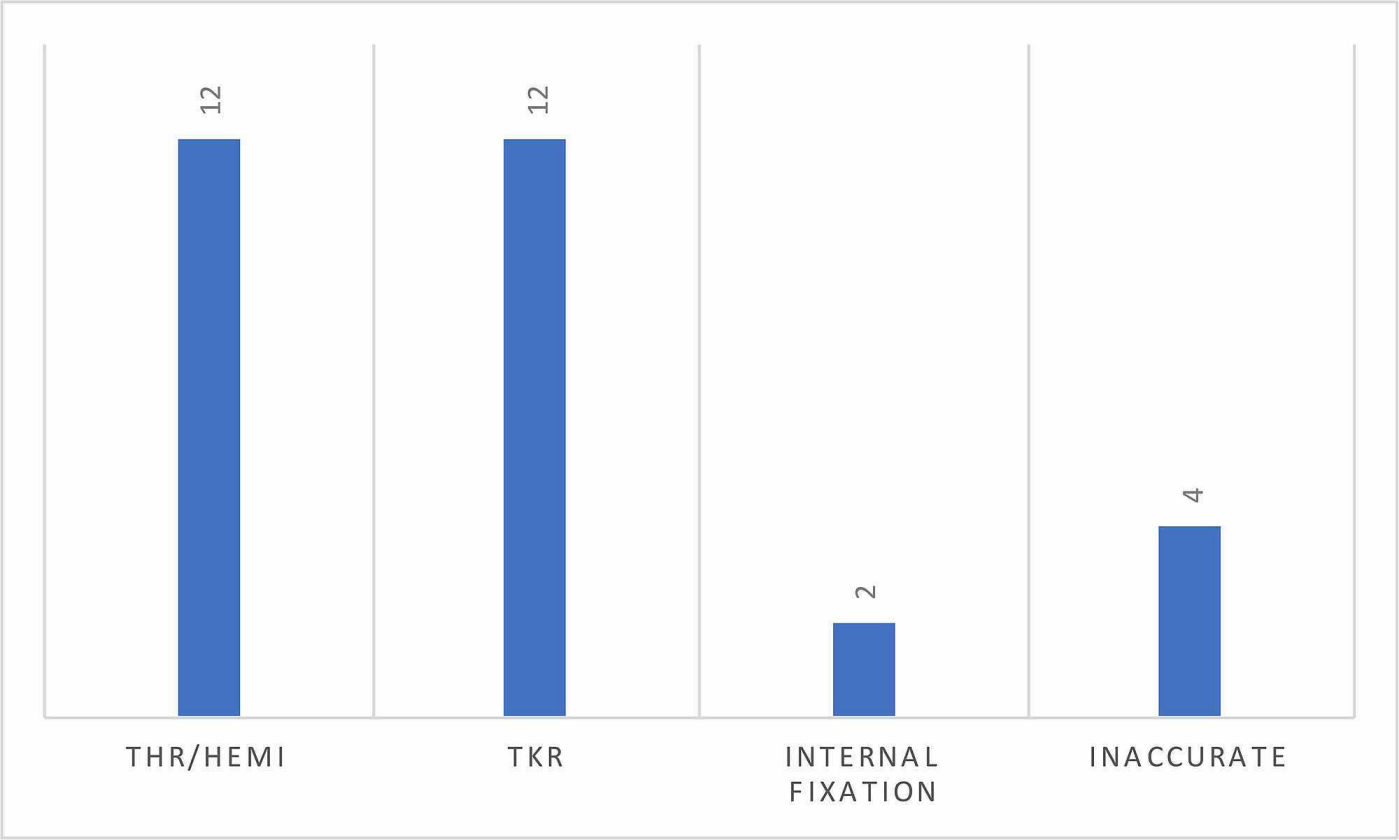
Fractures in relation to adjacent prosthesis. THR: total hip replacement; TKR: total knee replacement.

**Figure 2:**
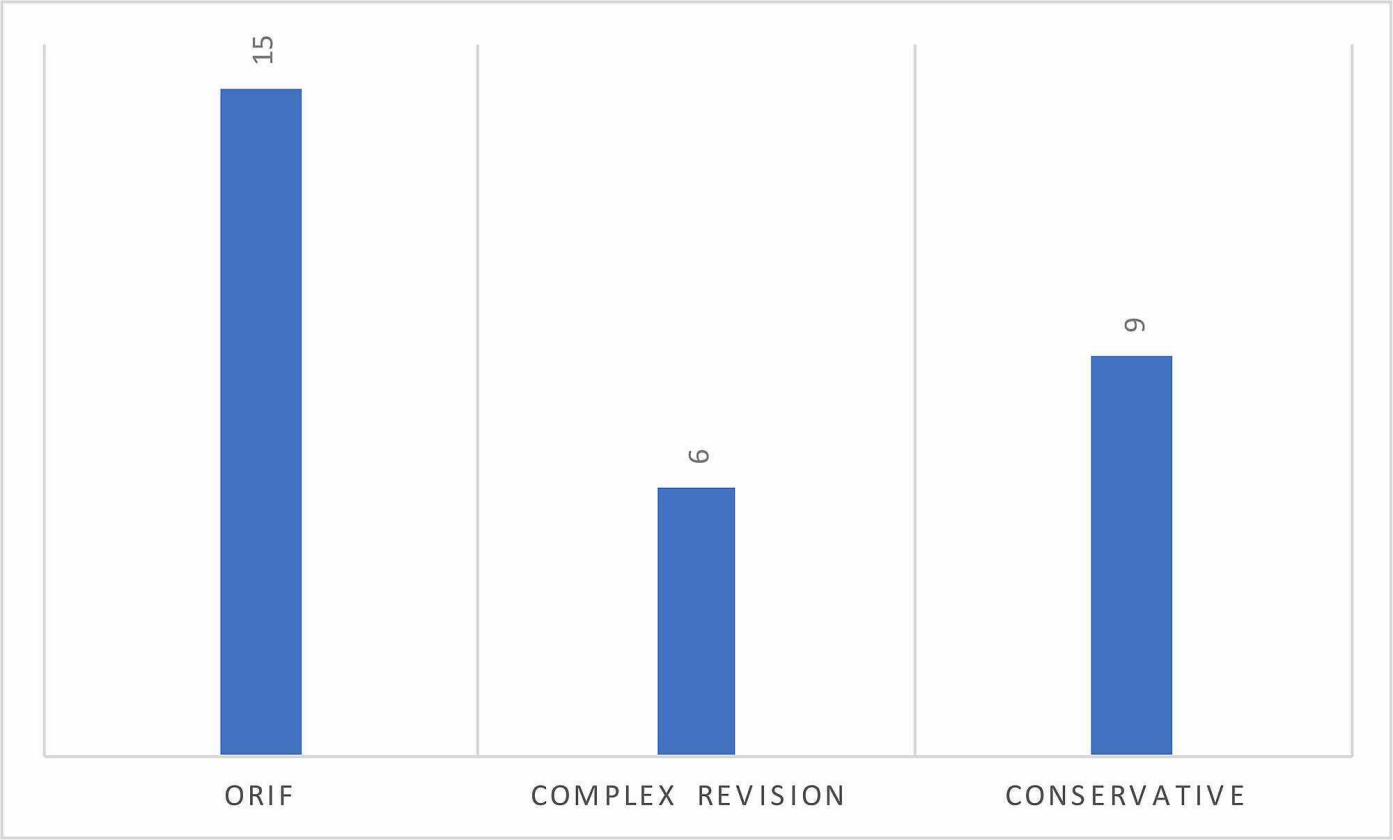
Types of surgery. ORIF: open reduction and internal fixation.

Using the M966 code, 29 patients with the diagnosis of periprosthetic fracture were identified by the coding team. A further patient was identified by reviewing operating theatre software (ORMIS®). Therefore, a total of 30 patients were identified, of which, there were 20 females and 10 males. Periprosthetic fractures included 12 fractures around total (THR) and hemi-hip replacements. Twelve fractures around total knee replacements (TKR) and two fractures around previous internal fixation devices (Figure 1). There were four patients who did not sustain periprosthetic fractures, accounting for 13.3% “correct diagnosis” error rate, these included one failed dynamic hip screw, one longstanding failed TKR, one failed tibial plateau ORIF, and one pubic ramus fracture with an ipsilateral long intramedullary nail treated conservatively. In terms of management of periprosthetic fractures, treatment consisted of ORIF, complex revisions and conservative management (Figure 2). 

Overall, excluding the patients who were managed conservatively, coding accuracy for surgically managed patients was 52% (n=21). This resulted in an estimated incurred loss of around £25,000 (Table 3). Wrong or omitted site of surgery was found to be the most common coder error with up to £8,000 loss in one case. 

**Table 3:** Losses.

Losses	
Clinician losses due to lack of co-morbidities documentation	£5,560
Coder losses due to error	£28,947
Reclaimed losses through this study	£10,050
Total incurred losses	£24,457

On the other hand, clinicians’ key contributing factors to inaccuracies in the coding process consisted of lack of documentation for comorbidities in the medical notes which lead to under coding and inaccurate (CC) scoring by the hospital coders. 

**Figure 3:**
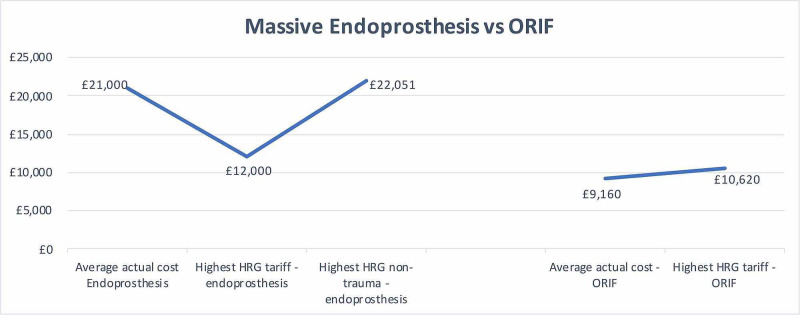
Massive endoprosthesis vs ORIF. ORIF: open reduction and internal fixation; HRG: Healthcare Resource Group.

Whilst analyzing the HRG tariffs, it was discovered that the highest possible HRG tariff that can be achieved when using a massive proximal or distal femoral endoprosthesis (HT12A) in a trauma setting would generate £12,000. In comparison, the same femoral endoprosthesis in a non-trauma setting (HN80A) equates to £22,051 (Figure 3). 

Overall, ORIF was associated with less financial loss to our trust with closer procedural costs to HRG tariff (average cost of £9,200 for ORIF vs £22,030 for a massive endoprosthesis) [3]. Using the findings of this study, approximately £12,000 was retrieved by the coding department for the trust. This was possible as coders have a two months window to make coding changes prior to final submission to the finance department.

## Discussion

Fractures around orthopaedic implants, such as internal fixation devices and joint replacements are classified as periprosthetic fractures and commonly result from low energy trauma. These occur predominantly around the hip and knee but can involve any bone with an orthopaedic implant [7]. Periprosthetic fracture management is highly complex requiring an experienced surgeon, with skills in revision surgery. The increased cost of these revision operations represents a significant financial burden to any healthcare systems worldwide [8,9]. Massive endoprosthesis is utilised when there is extensive bone loss from previous revision surgery, aseptic or septic loosening, fractures, or in orthopaedic malignancy cases [10].

Periprosthetic fractures are common amongst elderly patients who frequently have additional medical comorbidities adding to the complex nature of these fractures [1]. Periprosthetic fractures can result in significant morbidity and mortality. A study by Bhattacharyya et al. found one-year mortality as high as 11% in patients presenting with periprosthetic fractures [11]. High equipment and implant cost, lengthy surgery and ongoing social and rehabilitation care of these patients make this an expensive undertaking for hospitals [12]. Previous studies have found the length of stay has the largest financial impact [7,12] whereas, other studies have found long operating times due to the more technical nature of these fractures and optimising patients’ comorbidities pre-operatively through investigations and anaesthetic assessments as the most costly [13-15]. In addition, periprosthetic infection significantly added more to the costs [16]. 

The process of coding has many aspects to consider and is not always a straightforward task. The coding team is typically consisting of non-clinical staff. The title of the procedure in the operation note does not always translate to codes easily. The hospital coders rely on accurate documentation in patient notes, including admission documents, operating notes, and discharge summaries. Hospital coders cannot retrieve information from other sources such as old notes or General Practitioner (GP) letters for important information such as medical comorbidities and medications. Therefore, complete and accurate medical clerking by the admitting team is essential for hospital coders to accurately record comorbidities required for HRG tariffs and to reduce errors. From our analysis, we found coding errors from both the coding department and clinical teams. However, the cost implications to the trust were more financially significant due to coders errors when compared to the losses due to omission of medical comorbidities by clinicians. The hospital coders were consistent at coding for what is documented but were unable to select the correct code for surgical procedures occasionally. The most common errors were the omission or recording of wrong site of surgery, followed by lack and/or inadequate recording of co-morbidities by the admitting clinical team. The site of surgery is particularly significant in the final costing for these complex operations. For example, should a coder omit the term ‘femur’ for an ORIF procedure of a patient with an M966 code, this can result in a substantial underpayment of approximately £8,000 for the trust.

An important observation from this study was that the HRG tariffs differ on whether a revision procedure is performed in a trauma or an elective setting. We believe current HRG tariffs do not provide true reflection for the costs of these complex procedures. The highest possible HRG tariff that can be achieved when using massive femoral endoprosthesis (HT12A) in trauma would generate £12,000 compared to around £22,000 when the same prosthesis is used in an elective setting [3]. The cost of implants and equipment needed for such procedures is not covered by HRG tariffs under the umbrella of trauma (Table 2). If these operations could be re-categorized to an elective surgical procedure, there could be a substantial reduction in losses. In contrast, the tariff set for ORIF for periprosthetic fractures is in line with the highest HRG tariff for the procedure (£9,200 vs £10,600, respectively). This implies that should the surgeon decide to carry out an ORIF for periprosthetic fracture as opposed to selecting for a massive femoral endoprosthesis the cost savings for the trust would be significantly greater. However, this might not necessarily be the correct option for the patient. Moreover, patients who require revision procedures in a trauma scenario are often more medically unfit with often a longer length of stay potentially justifying the need for increased payment to institutions catering for these select group of patients. 

This discrepancy in tariff also raises the question should individual funding requests be considered when carrying out massive endoprosthesis procedures? As these procedures can be complex, requiring specialist equipment, most hospitals might not have all the required kit to deal with these complex procedures. This could result in extra cost via opting for loan kits from other trusts or medical device companies. One option could be that these complex procedures are outsourced to specialist centers, where all resources are available in order to minimize the total costs. The British Orthopaedic Association alongside British Hip and Knee Societies have recently released some guidance on managing such complex procedures in regional centres “Revision Hubs” only [17]. The main indication behind this is to allow such procedures to be done by surgeons who do them most frequently and by doing so produce better outcomes. However, there is also an argument that certain district general hospitals cover a large catchment area and it can be difficult for patients to be transferred to another hospital whilst they have a fracture.

From our study perspective performing more operations can in theory allow such specialist centers to negotiate better prices for implants from manufacturers and save on loan kits which therefore should reduce the overall costs in the long term. However, in certain district hospitals with big catchment areas such as ours, catering for a primarily elderly cohort of patients would be extremely challenging. Any delay to surgery can also increase the risk of developing complications such as pneumonia, pressure sores, deep vein thrombosis and pulmonary embolus. This would place the patient at a high risk of mortality having already sustained a significant injury.

We recognize this body of work has a number of limitations, which include, relatively low number of surgically managed patients, data collected from a single center and that the study sample was initially provided by hospital coders who could have potentially excluded patients who were coded incorrectly. However, to our knowledge, this is a first study looking into the financial impact of coding errors in the management of complex periprosthetic fractures and highlights the discordancy in payment between elective and trauma management of periprosthetic fractures.

While the coding errors can be easily rectified by adopting better coding practices and the use of technology to minimize human translation errors. The change necessary to address the discrepancy in HRG tariffs requires a complete overhaul of the HRG coding system. Fundamentally, we advocate re-evaluation of current HRG tariff rates by NHS commissioners, NHS Improvement and the Department of Health to ensure similar payment regardless of revision procedures being carried out in an elective or trauma setting. This would help trusts to recuperate potential losses for providing care for the management of highly complex periprosthetic fractures.

## Conclusions

Inaccurate coding of complex periprosthetic fractures leads to loss of income to our trust providing this essential service. Clinicians also have a role to play by ensuring accurate medical records from the time of admission till discharge. Coders need to be aware of the impact of omitting the site of surgery during the coding process. When in doubt, such procedures should be discussed individually with the surgical team to ensure the most accurate tariff is achieved. ORIF results in fewer losses in comparison to carrying out a massive endoprosthesis replacement. However, we do not support decision-making to be made purely on a financial basis. We believe clinicians should make their decisions on what would be best for patient in the short and long terms. Finally, HRG tariffs need re-evaluating and special measures need implementing to minimise financial losses so that centres can utilise resources efficiently and continue to provide the best care for all patients.
